# Nicotinamide Riboside Preserves Ovarian Injury in an Experimental Sepsis Model in Rats

**DOI:** 10.5152/eurasianjmed.2023.22255

**Published:** 2023-06-01

**Authors:** Jale Selli, Dilek Vural Keles, Osman Nuri Keles, Muhammet Celik, Zeliha Yetim

**Affiliations:** 1 Department of Histology and Embryology, Alanya Alaaddin Keykubat University Faculty of Medicine, Antalya, Turkey; 2 Department of Nursing, Kırklareli University Faculty of Health Sciences, Kırklareli, Turkey; 3 Department of Histology and Embryology, Atatürk University Faculty of Medicine, Erzurum, Turkey; 4 Department of Medical Biochemistry, Atatürk University Faculty of Medicine, Erzurum, Turkey; 5 Department of Histology and Embryology, University of Health Science Hamidiye International Faculty of Medicine, İstanbul, Turkiye

**Keywords:** Nicotinamide riboside, ovarian injury, oxidative stress, sepsis

## Abstract

**Objective:**

The aim of the study is to investigate the protective effects of nicotinamide riboside on oxidative stress in an experimental sepsis model created by cecal ligation and puncture.

**Materials and Methods:**

Rats were divided into 3 groups randomly: sham-operated (control) group, sepsis group, and nicotinamide riboside-treated group. Sepsis model-induced cecal ligation and puncture was applied to sepsis group rats. Animals in the nicotinamide riboside-treated group were administered nicotinamide riboside intraperitoneally (500 mg/kg). Tissue specimens from rats were biochemically calculated for their activities of catalase, superoxide dismutase, glutathione peroxidase, myeloperoxidase, and malondialdehyde levels. Ovarian tissues of all rats were histopathologically evaluated.

**Results:**

Catalase, superoxide dismutase, and glutathione peroxidase activities were lower in the sepsis group compared to the sham-operated (control) group. Superoxide dismutase activity was significantly higher in the nicotinamide riboside-treated group than in control and sepsis group (*P* < .05). Myeloperoxidase activities and mean malondialdehyde concentration of ovarian tissue were lower in nicotinamide riboside-treated group than in sepsis group (*P* < .05). The light microscopic assessment revealed that ovarian tissue was protected, and inflammation and interstitial edema decreased in nicotinamide riboside-treated group. The follicular damage findings were notably decreased in nicotinamide riboside-treated group in comparison to sepsis group (P<0.05).

**Conclusion:**

Our findings indicated that nicotinamide riboside diminished ovarian injury in sepsis via inhibiting tissue infiltration and increasing endogenous antioxidant capacity. Nicotinamide riboside administration may represent a new treatment approach for the prevention of sepsis-induced ovarian injury.

**Main Points**

- Sepsis is closely associated with multiple organ dysfunctions via inflammation.
- Reactive oxygen species of oxidative stress during sepsis affect ovary tissue.
- Nicotinamide riboside, a precursor of the nicotinamide adenine dinucleotide (NAD+), is a form of vitamin B3 and is present in a wide variety of our daily foods, such as milk, milk-derived products, and yeast.
- Nicotinamide riboside ameliorates sepsis injury by repairing the antioxidant system and, therefore, seems to be beneficial for overcoming inflammation in sepsis

## Introduction

Hyperinflammation in sepsis is closely associated with multiple organ dysfunction. According to previous studies, mechanisms that cause multi-organ damage seem to be associated with the pathogenesis of sepsis, which induces the development of apoptosis through inflammation-triggered cytokine activation and reactive oxygen production, mitochondrial pathway.^1^ An imbalance between the antioxidant defense system and emerging reactive oxygen species (ROS) results in the development of oxidative stress during sepsis. The endothelial cell–leukocyte interaction causes endothelial damage at the injection site, which results in the loss of endothelial integrity and tissue perfusion alteration.^2^ Also, the formation of microthrombi in the vessels activated by the inflammatory response causes microvascular dysfunction, which is a significant mark of organ damage in sepsis. The cytokines have more versatile roles as ovarian regulators in ovulation, folliculogenesis, and ovarian hormone synthesis. In addition, cytokines associated with bacterial endotoxins lead to disruptions in ovarian function and hormonal regulation and affect fertility indirectly mechanism.^3^ The cytokines such as IL-1 and TNF-α may affect the production of androstenedione and estradiol from theca and granulosa cells and inhibit the progesterone secretion from corpus luteum.^4^ Furthermore, experimental sepsis studies highlight increased malondialdehyde levels (MDA) and myeloperoxidase (MPO) activity and reduced glutathione (GSH-Px) levels in the ovarian tissue as the significant finding of oxidative stress.^5^ In the literature, it has been noted that sepsis-induced oxidative stress may result in the depletion of antioxidant reserves in ovaries.^5^ Therefore, it is logical to try to inhibit the degenerative effects of oxidative stress in ovary, which is an essential source of organ injury in sepsis.

Nicotinamide riboside (NR) is a form of vitamin B3 and is present in a wide variety of our daily foods, such as milk, milk-derived products, and yeast.^6^ Nicotinamide riboside has attracted attention as a precursor of the nicotinamide adenine dinucleotide (NAD+). The administration of NR productively improves intracellular NAD^+^ levels, a primary regulator of cellular oxidative stress, and may trigger sirtuins (SIRT) and poly (ADP-ribose) polymerases (PARPs) reactions.^7^ Recent studies have shown the effects of NR in different pathologies as well as in inflammatory conditions, mitochondrial disorders, metabolic syndrome, fatty liver disease, DNA repair syndromes, and Alzheimer's disease.^8^ Also, NR was shown to be successful in microvascular function improvement and attenuated endothelium damage in the ischemia-reperfusion model.^9^ As decreasing levels of NAD^+^ in sepsis have been reported in numerous studies, NR administration may be helpful in sepsis.^10^

In briefly, in this study, we aimed to investigate whether NR administration protects against oxidative stress and ovarian damage in a cecal ligation and puncture (CLP)-induced sepsis model.

## Materials and Methods

### Animals and Experimental Design

The present study was conducted according to national and international guidelines for using experimental animals. The protocols of experiments were reviewed and approved by the local and governmental committees for animal care and use (Ataturk University Local Ethics Committee, Aproval No: 2022-228). In total, 24 sexually mature female rats (Ataturk University Experimental Laboratories, Sprague Dawley, bodyweight 210-230 g) were used for all experiments in this study.

The rats were divided into 3 groups (n = 8): (a) sham-operated control group, (b) sepsis group (CLP + saline), and (c) nicotinamide riboside-treated group (CLP + NR) group. The animals in each group were kept in different cages. Cages were individually ventilated with a 12-hour light/dark cycle and maintained at 22 ± 2°C. Standard rat food and tap water were given ad libitum.

Sepsis was induced by the CLP technique. Briefly, rats were anesthetized (general anesthesia) with a mixture of ketamine (80 mg/kg) and xylazine (10 mg/kg) intraperitoneally (i.p.). The abdominal skin was shaved and disinfected, and the cecum was exposed by making a 2 cm midline incision. The cecum was joined with 4.0 silk sutures at 1/3 of the proximal cecum and punctured 3 times with an 18 G needle and gently tightened to remove stool from the perforation site. After the cecum was repositioned, the laparotomy was closed with 4.0 silk sterile sutures. All animals were resuscitated by subcutaneous injection of saline (30 mL/kg) after CLP. Thirty minutes before and 12 h after the CLP surgery, rats were treated with an intraperitoneal injection of high-dose NR (500 mg/kg) in the CLP + NR group or the same amount of saline in the CLP + saline group. The administered dose was used as it could ameliorate sepsis-induced multi-organ dysfunction.^11^ Nicotinamide riboside was obtained from BLD Pharmatech Ltd. (Cas: 23111-00-4, Shanghai, China) and dissolved in saline before use. The sham-operated group did not perform CLP, and other surgical procedures were the same as those of the CLP + saline group.

All CLP procedures were performed by the same investigator to minimize experimental variability. Twenty-four hours after CLP surgery procedure and respective treatments, rats (6 in the sham-operated group and 8 in each of the CLP + saline group and the CLP + NR group) suffered an assisted painless death with thiopental overdose (0.5 g/kg, i.p.) followed by decapitation. This time point was selected according to the fact that cytokine storm occurs between 12 and 16 hours after sepsis is created by CLP as reported in the literature.^12^ The ovaries were quickly removed; half of each ovary was washed with ice-cold saline and stored at −80°C for further biochemical analysis. The other half of ovary was fixed in a 10% formalin in order to analyze histopathological alterartions.

### Tissue Sample Preparation and Homogenization

For blind biochemical studies, ovarian tissue samples were labeled as groups 1, 2, 3, 4, etc., placed in liquid nitrogen promptly, and carried to the laboratory to measure lipid peroxidation levels and antioxidant and inflammatory enzyme activities. For the preparation of the ovarian tissue homogenates, all tissue samples were ground with liquid nitrogen in a mortar and then homogenized using a tissue homogenizer (Qiagen TissueLyser LT, Berlin, Germany) at 35 Hz (15 minutes at 4°C). The obtained homogenates were centrifuged at 1000 g for 10 min, and the supernatants were saved for analysis.

### Assays for Tissue Lipid Peroxidation and Antioxidant Enzymes

As explained previously, lipid peroxidation levels in ovarian tissue homogenates were measured spectrophotometrically according to Okhawa et al.^13^ The ovarian tissue samples were weighed as 15 mg in a sterile tube and homogenized in the cold with 1.5 mL buffers consisting of 100 g/L potassium chloride. The homogenates were centrifuged at 4°C, 10 000 g for an hour. Then, 0.25 mL of tissue homogenate was added to a solution containing 0.2 mL of 80 g/L sodium lauryl sulfate, 1.5 mL of 200 g/L acetic acid, 1.5 mL of 8 g/L TBA aqueous solution, and 0.3 mL of distilled water. The stirred mixture was heated in a boiling water bath for 45 minutes. After being cooled, 4 mL of n-butanol was added and layered by centrifugation. The absorbance of the supernatant was measured at 532 nm using a spectrophotometer (Bio-Tek, Winooski, VT, USA). 1,1,3,3 tetrametoxypropane was used to obtain the standard curve. Results were expressed in the unit of nanomoles per gram of tissue (nmol/g tissue). The superoxide dismutase (SOD) activity measurement was assigned according to Sun et al^14^ and catalase (CAT) activity according to Aebi et al.^15^ Estimation of the SOD activity was based on superoxide radical generation which was converted by the xanthine–xanthine oxidase system, the mentioned system reacts with nitroblue tetrazolium (NBT) for formazan dye creation. The assay of SOD activity was measured by NBT reduction method and the output was expressed as millimole per minute per milligram of tissue.

Catalase activity assay is based on the consumption of H2O2. For measuring CAT activity, the sample of supernatant was mixed with an H2O2 solution in phosphate buffer and measured in a quartz cuvette at 240 nm. Catalase activity was described as the amount of enzyme required to decompose 1 nanomole of H2O2 per minute, and results were expressed as mmol/min/mg.

The GSH-Px activity was evaluated in accordance with Paglia et al.^16^ The reaction in a tube was initiated with the addition of H202 and spectrophotometer was used to monitor the absorbance changes. Obtained results were expressed as mmol/min/g of tissue.

### Assay of Myeloperoxidase Enzyme for Polymorphonuclear Leukocyte Response

The modified method of Bradley et al^17^ was performed to determine MPO activity. Around 100 μL of the supernatant was added to 1.9 mL of 10 mmol/L phosphate buffer, and the homogenized tissue mixture (15 mg tissue in 1.5 mL buffer) was centrifuged for 10 minutes at 4°C. Myeloperoxidase absorbance changes were recorded at 460 nm every 30 seconds for 3 minutes after adding a solution that contained 1 mL of 1.5 mmol/L o-dianisidine hydrochlorides consisting of 0.0005% (w/v) hydrogen peroxide. The unit of MPO activity was defined as the amount required to degrade 1 μmol H2O2/min at 25^o^C. Results were expressed as units per milligram of tissue.

### Histopathological Examination

Ovaries were collected from the rats and immediately fixed in 10% buffered formalin for 24-48 hours. Before the tissue processing protocol, all tissues were washed in running water for 20 minutes and formalin was removed. For the tissue processing protocol, all tissues were dehydrated in a graded alcohol series and cleared by xylene series. At the end of the histological processing, the tissue samples were embedded in paraffin wax. After tissue processing, tissue blocks were sectioned at 5μm thickness with a microtome (Leica RM2125RT, Leica Instruments, Nussloch, Germany). For staining, following deparaffinization and rehydration procedures, sections were stained with hematoxylin and eosin (H&E). All group sections were observed under a light microscope (Nikon Eclipse 80i), and the slides' photomicrographs were taken using a digital camera.

### Data Analyses

In the present study, Statistical Package for Social Science (SPSS) version 20.0 for windows (IBM SPSS Corp.; Armonk, NY, USA) was used for statistical analysis, and descriptive statistical data were expressed as means ± standard error of mean. The significance of differences among different groups was analyzed by Duncan's multiple comparison test and one-way analysis of variance test. *P* < 0.05 was considered statistically significant.

## Results

### Biochemical Results

**Figure 1.**
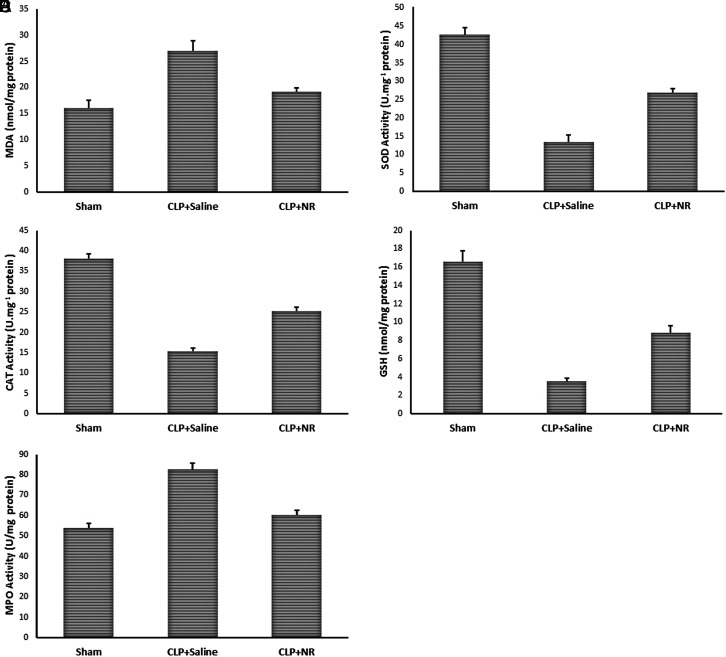
(A): The bar graph shows the mean level of MDA. (B): The bar graph shows the mean level of SOD activity. (C): The bar graph shows the mean level of CAT activity. (D): The bar graph shows the mean level of GSH. (E): The bar graph shows the mean level of MPO activity. CAT, catalase; SOD, superoxide dismutase; GSH, glutathione peroxidase; MPO, myeloperoxidase; MDA, malondialdehyde.

Results on ovarian tissue lipid peroxidation and antioxidant and immune response parameters during polymicrobial sepsis in high-dose NR-treated groups are shown in Figure 1A-E. High-dose NR inhibited oxidative stress associated with sepsis in ovarian tissue. The antioxidant enzymes SOD, CAT, GSH, leukocyte-derived enzyme MPO, and lipid peroxidation product MDA were detected 24 hours after the surgery modeling.

The ovarian MDA value was determined at the physiological level in the sham group (Figure 1A). The ovarian activities of SOD (69% and 37%, respectively), CAT (60% and 34%, respectively) enzymes, and the level of GSH (79% and 47%, respectively) were significantly lower in sepsis according to NR-treated group (Figure 1B-D). While the levels of MDA (68%) were significantly increased (Figure 1A), administration of NR significantly suppressed the MDA elevation levels (27%) in ovarian sepsis injury. Also, the activities of CAT, SOD, and GSH enzymes were significantly increased (101%, 65%, and 149%, respectively) in the CLP + NR group ovarian tissue, compared to the CLP + saline group (Figure 1B-D).

The lung MPO activities, an enzyme secreted by macrophages and activated neutrophils, were determined at the physiological level in the ovaries of the sham group (Figure 1E). High-dose NR treatment considerably decreased MPO activity that had been raised in the ovary by sepsis. The activities of the MPO enzyme were significantly increased (68% and 12%, respectively) either in the CLP + saline and in the CLP + NR groups. After NR treatment, the elevation in MPO activities (27%) was significantly lower in the CLP + NR group, compared to the CLP + saline group (Figure 1E).

### Results of Histopathologic Investigations

**Figure 2.**
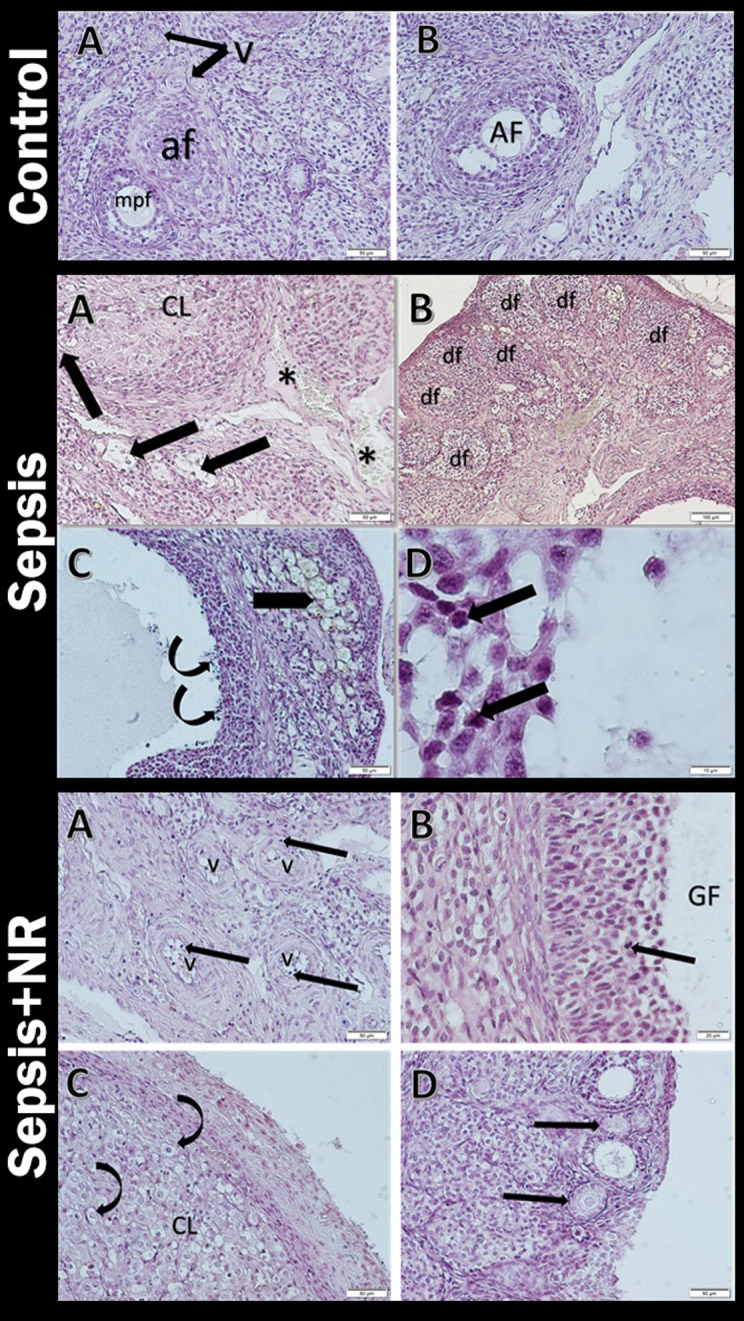
Control group: Representative photomicrographs of ovarian histology of sham-operated control group (A, B). af indicates atretic follicle; mpf, primary follicles with multiple layers; v, blood vessels; and AF, normal antral follicle. H&E staining; original magnification ×40. Sepsis group: Representative photomicrographs of ovarian histology of sepsis group (A, B, C, D). (A) shows corpus luteum (CL), cytoplasmic vacuolization (black arrow) of granulosa cells, vascular damage, and edema (*) in the stroma. (B) includes many degenerated (atresia) follicles (pdf). (C) shows apoptotic bodies (crooked arrow) in the Graafian follicle and widespread vascular damage (black arrow) in the stroma. (D) exhibits degenerative cells with nuclear changes (black arrows) consisting of pyknosis and karyorrhexis in the Graafian follicle. H&E staining, original magnification A (×40)—B (×20)—C (×40)—D (×100). Sepsis + NR group: Representative photomicrographs of ovarian histology of NR group. (A) shows blood vessels with normal morphology in the medulla of the ovary, and few leukocytes (black arrow) were observed. (B) exhibits theca and granulosa cells of the Graafian follicle (GF) with normal cellular structure and few leukocytes (black arrow). (C) demonstrates vacuolization in some cells of the corpus luteum (convoluted arrow). (D) shows healthy growing follicles (black arrow). H&E staining, A(×20)—B(×40)—C(×40)—D(×20)).

Sham-operated (control) group ovaries exhibited a typical healthy appearance with the cortex and medulla, and no damage was observed in the ovarian tissue (Figure 2). The germinal epithelium surrounding the ovary is observed as a simple squamous epithelium. Many primordial, preantral, and antral planned follicles were located in the cortex and the medulla. Corpus luteums were marked slightly acidophilic.

There were severe inflammation and interstitial edema in CLP + saline group ovarian tissue samples. The pathological changes, such as massive neutrophil infiltration, increased vascular permeability, and tissue edema, primarily vascular endothelial damage, were conspicuous in this group's ovarian medulla (Figure 2). Sepsis caused an increase in necrotic and apoptotic cell numbers due to decline in granulosa cell viability. Cytoplasmic vacuolization was available in the luteinized granulosa cells of the corpus luteums and the granulosa cells of the primary and secondary follicles (Figure 2). Apoptotic and degenerative cells with nuclear changes consisting of hyperchromatic nuclei, pyknosis, and strongly eosinophilic cytoplasm were also visible in those kinds of follicles (Figure 2). However, there were no striking differences in the early stages of follicles. In addition, many atretic cells in the Graafian follicles and hypertrophic cell changes in the early-stage follicles were observed in sepsis-induced ovarian samples compared to the sham-operated group (Figure 2).

Nicotinamide riboside treatment significantly reduced histopathological changes due to sepsis and preserved ovarian structures. Although some leukocytes were seen in interstitial tissue of the ovarian sections of the CLP + NR group, dense inflammatory cell migration was significantly decreased compared to the CLP + saline group (Figure 2). Vascular structures were preserved, and edema was minimal, as NR provided an improvement in tissue architecture (Figure 2). Degenerative changes and apoptotic cell death in granulosa cells were very few compared to the sepsis group (Figure 2). Follicular development and generally follicular cell structure were similar to the sham-operated group (Figure 2).

### Inflammation Scoring

Inflammation scoring on histopathological investigations was made according to the grading system in Table 1. It was obvious that polymorphonuclear leukocytes, vascular congestion, and edema scores were lower in CLP + NR group compared to the CLP + saline group.

## Discussion

Numerous studies have described oxidative stress as significant event of sepsis,^18^ which may lead to cellular damage and organ dysfunction.^19^ Multiple generations of nitric oxide (NO) and ROS are the main sources of oxidative stress during sepsis.^18^ Reactive oxygen species may damage vascular endothelial cells; free oxygen radicals induce the expression of tissue factors and contribute to sepsis-induced microvascular dysfunction.^20^ Microvascular perfusion alterations and subsequent organ failure may develop due to septic conditions. Despite recent advances in therapeutic agents, organ failure in sepsis is still debated. As oxidative stress plays a critical role in initiating and establishing sepsis, supplementation with antioxidants seems beneficial and improves current sepsis therapies. For this purpose, we aimed to investigate the antioxidant effects of NR on ovarian damage in a CLP-induced sepsis model.

The activity of ROS scavengers like SOD and CAT considerably decreases in the tissues during sepsis.^20^ In the present study, we revealed oxidative stress damage through measurements of MDA, MPO, SOD, CAT, and GSH-Px enzymes, as important endogenous antioxidants against oxidative stress damage in ovary. Increased MDA and MPO levels and decreased SOD and CAT activities demonstrate sepsis-induced ovarian tissue damage in the present study. The data also support the preservative effect of NR in a sepsis model of tissue injury. The crucial finding in present study was that NR administration resulted in a significant decrease in MPO activity as an indicator for active leukocyte accumulation in tissue. This finding suggests that NR can potentially influence tissue injury and inflammation.^11^ Additionally, observed tissue recovery evidence supports this finding. Namely, the administration of NR in CLP-induced sepsis significantly decreased leucocyte infiltration and tissue edema and reduced apoptotic follicular cells by inhibiting oxidative stress.

Malondialdehyde is a typical product of oxidative damage and a good indicator of lipid peroxidation. Lipid peroxidation plays an essential role in membrane permeability changes, eventually leading to cell lysis.^21^ This study found that MDA levels in the ovarian tissue increased in CLP-performed septic rats. Elevated tissue MDA levels were described in CLP-induced sepsis models in rats and humans.^22^ Also, data from the present study exhibit that MDA content was high in ovarian tissue during sepsis; however, NR administration inhibited the MDA concentration increase in septic rats.

In a CLP-induced sepsis model, excessive ROS products such as superoxide (O_2_^−^) and H2O2 are metabolized by SOD, CAT, and GSH-Px, the major antioxidants that protect cytosolic organelles from toxic free radicals during oxidative stress.^11^ Similar to our results, recent studies reported that SOD and CAT levels decreased during CLP-induced sepsis in the affected tissues.^23,24^ Our data demonstrated that the administration of NR in the CLP septic group increased antioxidant enzyme activities. Glutathione peroxidase and SOD activity significantly improved in CLP+ NR group compared to CLP + saline group septic rats. These results suggest that NR attenuates ovarian tissue injury by preventing oxidative stress after CLP-induced sepsis. Nicotinamide riboside increased the antioxidant activity and attenuated inflammation and oxidative stress by activating macrophages.^25^ Recent study indicates that pre-administration of NR supplies anti-inflammatory and protective effects against apoptosis through the NAD^+^/SIRT1 signaling during the inflammation.^11^ Interestingly, SIRT1 activation was significantly lower due to decreasing NAD^+^ in sepsis.^26^ As NR has been shown to be considerably effective in raising NAD^+^ levels in humans^27^ and experimental models,^11,28^ it was suggested that NR may be useful in sepsis injury.

Apoptotic cell death of various tissues under sepsis conditions has been described in the literature.^29^ Kafa et al^30^ declare that the number of apoptotic cells in different brain regions significantly increased in CLP-induced sepsis model. In addition, cytoplasmic blebs, necrotic cells, apoptotic bodies, and cell membrane damage were shown in the murine sepsis model.^29^ A recent study similar to our results^31^ described an increased number of apoptotic cells, mainly in the Graafian follicles in the CLP-induced sepsis model. As excessive ROS production leads to oxidative tissue damage in the ovary, pharmaceutical agents should demonstrate an antioxidant effect to reduce ovarian tissue damage.^32^ The proper antioxidant dosage of NR to initiate tissue protection has not been described in the literature. In experimental animal studies, the dosage of NR has varied between 100 and 500 mg/kg body weight.^11,33^ We administered 500 mg/kg of NR for each rat to evaluate its antioxidant and anti-inflammatory effects on sepsis-induced injury. Nicotinamide riboside is a prescribed precursor of NAD^+^, which is an essential regulator of oxidative stress and inflammatory responses. Nicotinamide riboside decreases oxidative damage and diminishes brain inflammation, karyopyknosis, and degeneration in brain tissue.^34^ Also, present study results demonstrated that NR decreases leucocyte infiltration, tissue edema, degenerative changes, and apoptotic cell death in follicles. Our findings confirm Hong's study,^11^ which reported that inflammatory cell infiltration, tissue edema, and apoptosis were reduced by NR at 500 mg/kg in septic mouse lungs. The selected dose of NR in the present study was based on this study.^11^ We hope that mentioned properties of NR may be related to its effects on the apoptosis pathway and NF-kB as a main mediator of inflammation. Though, the limitation of our study is the lack of measurement of NF-kB, caspase-3 activity, and cytokine levels.

In conclusion, this study is the first experimental model investigating NR on ovarian injury. The present study's data have shown that NR's protective effect may be due to its potent antioxidant property, which causes improvements in oxidative stress parameters in ovarian tissue. Nicotinamide riboside ameliorates sepsis injury by repairing the antioxidant system and therefore, seems to be beneficial to overcome inflammation in sepsis. It has long been used as a dietary supplement. Also, it could become a treatment modality for oxidative stress in sepsis-induced organ injury if further studies support our results. Based on our findings, dietary supplementation of NR may ameliorate ovarian injury and have therapeutic effects as a preservative approach in sepsis. However, clinical trials with larger sample sizes are required to investigate the antioxidant effect of NR in sepsis injury and to achieve precious decisions.

The limitations of our study were given as follows:

1. This study also aimed to evaluate NR effect on CLP. However, we had no diagnostic kits for the measurement of anti-inflammatory and inflammatory cytokine levels and we could not do it.
2. We could not evaluate immunohistochemically on ovary tissue due to lack of immunohistochemical kit and antibody.

## Figures and Tables

**Table 1.** Sepsis Injury on Histopathological Inflammation Scores of Ovarian Tissues of Rats

Groups	PMNL	VK	Edema
Sham operated (control)	0	0	0
CLP + saline	3	3	2
CLP + NR	1	2	1

The grading system was described as 0, no inflammatory cells (ICs); 1, a few ICs; 2, many ICs in the peripheral parts of the perivascular area; 3, numerous ICs in the perivascular area.

PMNL, polymorphonuclear leukocytes; VC, vascular congestion.
